# Novel Resveratrol and 5-Fluorouracil Coencapsulated in PEGylated Nanoliposomes Improve Chemotherapeutic Efficacy of Combination against Head and Neck Squamous Cell Carcinoma

**DOI:** 10.1155/2014/424239

**Published:** 2014-07-09

**Authors:** Aarti Mohan, Shridhar Narayanan, Swaminathan Sethuraman, Uma Maheswari Krishnan

**Affiliations:** ^1^Centre for Nanotechnology & Advanced Biomaterials, School of Chemical and Biotechnology, SASTRA University, Thanjavur 613401, India; ^2^AstraZeneca India Pvt. Ltd. Avishkar, Bellary Road, Hebbal, Bangalore 560024, India

## Abstract

Increasing consumption of tobacco and alcohol has led to a steady increase in the incidence of head and neck cancers in Asia. The drawbacks associated with the existing chemotherapeutic and surgical interventions have necessitated the development of a safer alternative for therapy of head and neck cancers. In this study we have explored the synergistic therapeutic potential of a phytochemical and chemotherapeutic agent using PEGylated liposomes as a delivery vehicle. Resveratrol and 5-fluorouracil were successfully coencapsulated in a single PEGylated nanoliposome. The thermal analysis and the nuclear magnetic resonance results revealed that resveratrol localized near the glycerol backbone of the liposomal membrane while 5-fluorouracil localized closer to the phosphate moiety, which influenced the release kinetics of both drugs. The nanoformulation was tested * in vitro* on a head and neck cancer cell line NT8e and was found to exhibit a GI_50_ similar to that of free 5-fluorouracil. Further, gene expression studies showed that the combination of resveratrol and 5-fluorouracil exhibited different effects on different genes that may influence the net antagonistic effect. The coencapsulation of resveratrol and 5-fluorouracil in a liposomal nanocarrier improved the cytotoxicity in comparison with the free drug combination when tested * in vitro*.

## 1. Introduction

The use of combination chemotherapy along with surgery or radiotherapy has been conventionally practiced to treat head and neck squamous cell carcinoma (HNSCC) [1, 2]. However, the toxic side effects of the conventional therapy remained which sometimes reduced the chances of remission. The therapeutic regimen mainly involves the surgical removal of the diseased area followed by adjuvant chemotherapy involving either cisplatin or 5-fluorouracil (5-FU) or both in combination with docetaxel or cetuximab along with radiation therapy [3, 4]. The side effects involve facial disfiguration and associated psychosocial trauma and difficulties in eating and in some cases even breathing, in addition to chemotherapy related side effects such as nausea, fatigue, alopecia, anemia, neutropenia, and mucositis [5, 6]. Hence an efficient therapeutic modality that can act as a substitute for surgery and minimize the chemo- and radiotherapy related side effects is the need of the hour.

In this context, use of biodegradable drug delivery vehicles such as liposomes would not only enhance the bioavailability of the drug and lower the concentration of the drug to be administered but also provide targeting options to the tumor cells [7, 8]. Liposomal formulations are available for conventional chemotherapeutic drugs like doxorubicin [9] and platinum derivatives [10, 11]. However, even the use of a nanoparticulate drug delivery system does not completely eliminate the toxic side effects [12, 13]. In order to address these issues, alternate therapies such as plant polyphenols are being explored for their anticancer effects [14, 15].* trans*-Resveratrol (Res) is one such polyphenol, belonging to the stilbenoid family, which has been extensively explored for its anticancer, anti-inflammatory, cardioprotective, neuroprotective, and antidiabetic effects [16–19]. Resveratrol has also been found to potentiate the efficacy of chemotherapeutic agents and minimize the side effects associated with them [20–22]. However, owing to poor bioavailability and rapid metabolism, resveratrol is considered only as a supplement and is not considered as a potent drug molecule [23, 24].

The combination of resveratrol and 5-fluorouracil has been tested* in vitro* in a number of cell lines and animal models [23–27]. However, all the studies were carried out using higher concentrations of both drugs, which are physiologically not possible to achieve at the disease site [23]. The concept of using nanoparticles to deliver two drugs simultaneously at the diseased site has garnered much attention since it enables targeting multiple molecular targets in the cell to annihilate cancer cells more effectively [28, 29]. However, such strategies remain unexplored for treatment of head and neck cancer. Moreover, the combination of resveratrol and 5-fluorouracil has also not been investigated for head and neck cancer therapy. Therefore the present work aims to evaluate the effect of this combination on a head and neck squamous cell cancer cell line and formulate a dual drug loaded PEGylated liposomal system encapsulating resveratrol with the chemotherapeutic drug 5-fluorouracil to achieve cell death via apoptosis at low concentrations of both drugs. Further, the nature of interaction of the two drugs and the signaling molecules regulated at the gene levels have also been studied in order to deduce the pathway involved in the mechanism for cell death with this combination.

## 2. Materials and Methods

### 2.1. Materials

L-*α*-Phosphatidylcholine (egg PC, EPC) was purchased from Avanti Polar Lipids, Alabaster, AL, USA, and distearoyl phosphatidyl serine-polyethylene glycol (2000) (DSPE-PEG-2000) was purchased from Northern Lipids, Canada.* t*-Resveratrol was a kind gift from Orchid Chemicals and Pharmaceuticals Ltd. (OCPL, Chennai, India) and 5-fluorouracil was purchased from Sigma Aldrich, India. Methanol, chloroform and dimethyl sulfoxide (DMSO) were all purchased from Merck, India. NT8e, a head and neck squamous carcinoma cell line, was a kind gift from Dr. Mulherkar's lab, Advanced Centre for Training, Research and Education on Cancer (ACTREC), Mumbai [30]. Dulbecco's Modified Eagle's Medium (DMEM), Fetal bovine serum and antibiotic (penicillin/streptomycin), and Trizol reagent were purchased from Gibco, India.

### 2.2. Methods

#### 2.2.1. Preparation of PEGylated Liposomes

Liposomes were synthesized by thin film hydration method. The lipids were dissolved in chloroform and made into a thin film by removing the chloroform in vacuum followed by hydration in phosphate buffered saline (PBS), pH 7.4 and stirring at 60°C for 45 minutes. The liposomal suspension was then extruded through polycarbonate membrane with pore size of 200 nm (Liposofast Basic, Avestin, Canada). In case of drug loaded liposomes, resveratrol dissolved in methanol was loaded in respective drug : EPC : DSPEPEG ratios (w/w) while 5-fluorouracil dissolved in PBS was loaded in the drug : EPC : DSPE-PEG ratio (w/w). Both drugs were passively loaded. The dual drug formulation was synthesized in the resveratrol : 5-fluorouracil : EPC : DSPE-PEG ratio of 1 : 1 : 18 : 2 (w/w). Unencapsulated resveratrol was removed by centrifugation at 4500 rpm for 15 minutes and drug loaded liposomes were separated from unencapsulated 5-fluorouracil by centrifugation at 16000 rpm for 45 minutes.

#### 2.2.2. Morphological Characterization

The liposomes were analysed for their morphology using field emission transmission electron microscope (FE-TEM) (JEM 2100F, JEOL, Japan). The liposomes were placed on the copper grid, air dried at room temperature, and subsequently imaged.

#### 2.2.3. Particle Size and Zeta Potential Analysis

Liposomal suspension was prepared in phosphate buffered saline solution (pH 7.4). Particle size and zeta potential analysis was performed using dynamic light scattering (Zeta Sizer, Malvern Instruments, UK). To determine the colloidal stability, the prepared liposomes were kept undisturbed at 37°C. 1 mL samples were aliquoted and analyzed for their size after 0, 6, 12, 18, and 24 hours.

#### 2.2.4. Thermal Analysis

Eight milligrams of each lyophilized sample was loaded in aluminum pans in the differential scanning calorimeter (Q20, TA Instruments, USA). The scans were carried out between 10°C and 100°C at a scan rate of 2°C per minute.

#### 2.2.5. Estimation of Encapsulation Efficiency

The extruded samples were separated from the unencapsulated drug as mentioned earlier. The liposome pellets were dissolved in methanol and used to determine the encapsulation of the drugs using a UV spectrophotometer (Lambda 25, Perkin Elmer, USA) after blank correction with plain liposomes. The resveratrol was detected at 306 nm while encapsulated 5-fluorouracil was detected at 265 nm. Absorbance was converted to amount of drug using a standard graph and the encapsulation efficiency was calculated using the following:

(1) $$\begin{array}{c} \text{Encapsulation}\;\text{Efficiency}\left(\%\right)=\frac{\text{Drug}\;\text{encapsulated}}{\text{Total}\;\text{Drug}}\times 100. \end{array}$$

#### 2.2.6. Release Kinetics

The dialysis bags (HiMedia, India) were immersed in distilled water for 1 hour at 60°C to remove any preservatives and subsequently rinsed with PBS. The liposomes were separated from the unencapsulated drug as mentioned earlier and the pellet containing drug loaded liposomes was resuspended in 200 *μ*L PBS (pH 7.4) and added into a dialysis bag, which was sealed on both ends. The dialysis bag was then immersed in buffer and the release kinetics was estimated in PBS (pH 7.4) and simulated saliva medium (pH 6.8) [31, 32] at 37°C. The sink volume was maintained at 30 mL and 1 mL aliquots were taken at the end of each time point and replaced with 1 mL of release medium. The aliquots were analyzed using UV spectrophotometer. The absorbance was converted into concentration using a standard graph and percentage release was calculated.

#### 2.2.7. Solid State NMR Analysis

The drug loaded liposomal formulations were prepared as mentioned earlier and subsequently lyophilized (Martin-Christ, Germany). The presence and localization of both drugs in the liposomal membrane were analyzed using solid-state ^31^P and ^13^C NMR (DSS spectra, 300 MHz Bruker, Germany). The analyses were performed at a spinning speed of 10 KHz. Phosphoric acid and L-glycine were used as standards for ^31^P and ^13^C analysis, respectively.

#### 2.2.8. Determination of GI_50_ for Drugs and Their Liposomal Formulations

NT8e oral squamous cell carcinoma cells were cultured in DMEM with 10% FBS and 1% antibiotic (penicillin/streptomycin) and maintained in 5% CO_2_ humidified chamber at 37°C. The cells were seeded at a density of 3000 cells/well in a 96-well plate, allowed to adhere overnight, and subsequently treated with a range of concentrations (0.0064 *μ*M to 100 *μ*M) of free resveratrol and 5-fluorouracil as well as their respective liposomal formulations and incubated for 48 hours at 37°C. After 48 hours, sulforhodamine (SRB) assay was performed [33]. The dye was solubilized in 10 mM Tris base and estimated at 564 nm using Tecan multimode reader (Infinite M200, Tecan, Austria). The GI_50_ of the drugs and their respective liposomal formulations were estimated using GraphPad Prism software, Version 6.

#### 2.2.9. Drug Combination Analysis Using Median Effect Principle

The nature of interaction of both drugs* in vitro* was determined by treatment of NT8e cells with different concentrations of resveratrol and 5-fluorouracil that were close to their respective GI_50_ values. The cells were seeded at a density of 3000 cells/well and allowed to adhere overnight. 5-Fluorouracil was administered in a concentration range of 0.25 *μ*M to 5 *μ*M and resveratrol was administered in a range of 3 *μ*M to 30 *μ*M and incubated for 48 hours in a humidified chamber at 37°C. After incubation, SRB assay was performed as described previously. All possible combinations of the two drugs within these concentration ranges were analyzed for any additive, synergistic, or antagonistic effects. The data were analyzed for combination index using Compusyn software, version 1.

#### 2.2.10. Gene Expression Analysis

The cells were treated with the GI_50_ concentration of drugs and their respective liposomal formulations for 48 hours after which the media were discarded and a gentle wash with sterile PBS was given. Then Trizol reagent was added to the cells and incubated for 30 minutes after which the extracts were collected. The mRNA extraction procedure followed by cDNA synthesis was carried out for each sample using the QuantiTect Reverse Transcription kit (Qiagen, USA). The PCR was carried out using the QuantiTect SyBr Green kit (Qiagen, USA) in an Eppendorf thermocycler. A ΔΔCT analysis was carried out using untreated cells as control. The primer sequences used for the gene expression studies are listed in Table 1.

### 2.3. Statistical Analysis

The experiments were performed in triplicate (*n* = 3) and they are represented as mean ± standard deviation. Data were analyzed using one-way ANOVA followed by Tukey test with the level of significance of *P* < 0.05.

## 3. Results and Discussion

### 3.1. Encapsulation Efficiency

Liposomal formulations for individual drugs were prepared using different drug to lipid ratios and encapsulation efficiencies were calculated. The PEGylated formulations for the individual drugs were optimized for the highest encapsulation efficiency, and using these values as reference, the dual drug loaded formulation was optimized. The dual drug loaded formulation was optimized in such a manner so as to obtain maximum encapsulation efficiency of both drugs when loaded together in the same PEGylated liposomal formulation. Finally, 1 : 1 : 18 : 2 was chosen as the optimized dual drug : lipid ratio, which had about 42% of resveratrol and 25% 5-fluorouracil.

Table 2 summarizes the encapsulation efficiencies obtained for 5-FU at different drug to lipid compositions. Surprisingly, introduction of PEGylated lipids decreased the encapsulation efficiency of 5-fluorouracil by 2.5 times when compared with its non-PEGylated counterpart. When 5-fluorouracil was coencapsulated with resveratrol, its encapsulation efficiency decreased when the resveratrol content was increased. A maximum encapsulation efficiency of about 15% was achieved at a ratio of 1 : 20 : 1 5-FU : EPC : resveratrol.

An increase in the lipid content and maintaining an equimolar concentration of 5-fluorouracil and resveratrol decreased the encapsulation efficiency. However, doubling the resveratrol content and lipid content while maintaining the 5-fluorouracil constant at a ratio of 1 : 40 : 2 5-fluorouracil : EPC : resveratrol restored the value for the encapsulation efficiency of 5-fluorouracil to 15%. Introduction of PEGylated lipids during coencapsulation of 5-fluorouracil and resveratrol was found to exhibit a positive effect on the encapsulation of 5-fluorouracil.

Table 3 shows the encapsulation efficiencies of resveratrol at different drug to lipid ratios. It is observed that the encapsulation efficiency of resveratrol is influenced by the amount of lipids in the formulation. It was found that the encapsulation of resveratrol was found to be maximum in the 1 : 40 drug : lipid ratio beyond which no significant improvement was observed in the encapsulation efficiency. Introduction of 5-FU in the case of dual drug loaded liposomes did not affect the encapsulation of resveratrol. A reduction in encapsulation of resveratrol is observed on introduction of PEGylated lipid in the formulation. This may be due to the enhanced hydrophilicity conferred to the liposomal surface by the PEG chains that may retard the encapsulation of resveratrol. Among the PEGylated liposomes, the encapsulation was decreased by 32% in the 1 : 38 : 2 combination (drug : egg PC : DSPE-PEG) when compared with the 1 : 18 : 2 (drug : egg PC : DSPE-PEG) ratio. Further addition of 5-FU in the formulation in a 1 : 1 ratio to resveratrol led to the increase in the encapsulation to 41.56 ± 0.695%. However, addition of 5-FU in the PEGylated formulation did not influence the encapsulation of resveratrol significantly. These differences in the encapsulation of resveratrol may arise due to a difference in the localization of resveratrol directed by the presence of PEG chains or 5-FU.

The encapsulation efficiency of a molecule in a liposome depends on its polarity and partition coefficient, which also determines its localization in the liposomal membrane. If a drug is hydrophobic in nature, it resides in the acyl hydrocarbon chain of the liposome. Hence the encapsulation is dependent on the properties of the acyl chains of the liposome such as length of the acyl chain and packing density and volume. On the other hand, if a drug is polar/hydrophilic, it tends to localize in the aqueous core or adjacent to the water-lipid interface of the liposome. Thus, it is expected that resveratrol, a hydrophobic molecule, will preferentially localize near the fatty acyl chains of the liposome and hence its encapsulation efficiency is influenced by changes in drug to lipid ratio. On the other hand, 5-fluorouracil, a polar molecule, tends to localize near the polar head groups of the liposome and hence its encapsulation efficiency does not exhibit a strong dependence on the drug to lipid ratio. Introduction of the hydrophilic chains in the liposome surface favors greater entrapment of the hydrophilic 5-fluorouracil when compared with resveratrol and this is reflected in the encapsulation efficiencies.

### 3.2. Morphological Characterization of Drug Loaded PEGylated Liposomes

**Figure 1:**
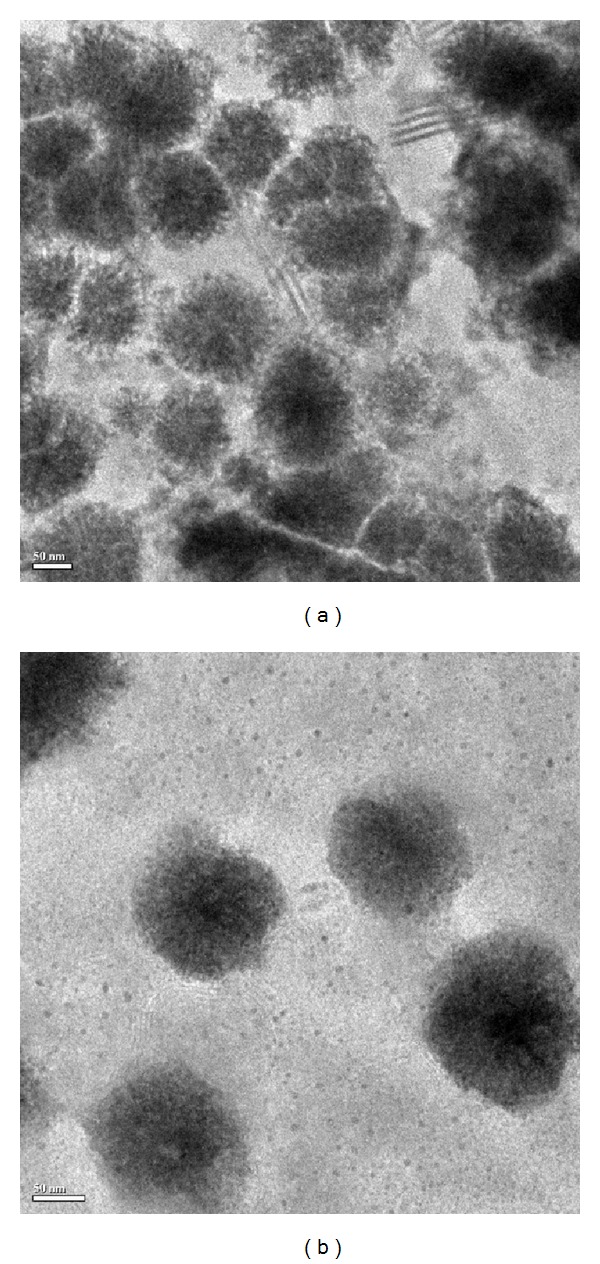
FE-TEM images of (a) blank PEGylated liposomes and (b) dual drug loaded PEGylated liposomes.

Figure 1 shows the high-resolution transmission electron micrographs of the dual drug (resveratrol and 5-fluorouracil) loaded PEGylated liposomes. Both blank and dual drug loaded liposomes were found to have a spherical shape (Figure 1).

**Figure 2:**
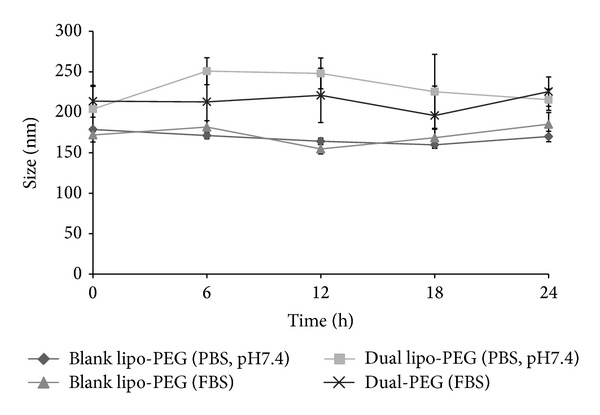
Particle size analysis of dual drug loaded PEGylated liposomes.

The size distribution of the dual drug loaded liposomes varied between 200 and 250 nm as measured using dynamic light scattering. The average particle size of blank and PEGylated dual drug loaded liposomes was 178 ± 4 nm and 204 ± 48 nm. Several groups have reported that liposomes of size 100 to 300 nm extravasate and localize in the tumor tissue. It has also been found that hydrophilic moieties like polyethylene glycol aid in circumventing clearance by the reticuloendothelial system [35–37]. The liposomes exhibited good colloidal stability and maintained their size for 24 hours in a protein-containing medium (Figure 2). Zeta potential is a measure of the potential difference between the stationary fluid phase around the nanoparticle and the dispersion medium, which reflects in the stability of the suspension [38]. Hence, in general, the values of zeta potential for stable nanoparticle suspensions should be either lesser than −30 mV or greater than +30 mV. Any value between this range shows that the particle has larger tendencies to aggregate leading to the sedimentation of the particles. In the present study, the zeta potential of the liposomal formulations was −39.50 ± 3.93 mV for blank liposomes and −29.50 ± 1.34 mV for the dual drug loaded liposomes indicating good stability. The 24 h particle size study also supported the zeta potential data, hence illustrating that the dual drug loaded PEGylated formulation was a stable colloidal suspension in PBS (pH 7.4) and in presence of 1% FBS.

### 3.3. Thermal Properties

**Figure 3:**
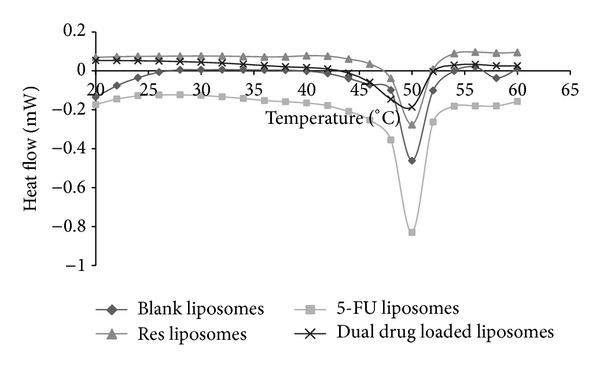
Heat flow profiles of PEGylated liposomes in the presence and absence of drugs.

Differential scanning calorimetry (DSC) was performed to study the thermal transitions in resveratrol-loaded, 5-FU-loaded, and dual drug loaded liposomes (Figure 3). These studies give an insight to the physicochemical interactions of the two molecules with the liposome membrane. It is known that the localization of the drugs in the liposomes influences their release properties and also the stability of the liposome [39]. It was observed that presence of resveratrol in the liposomes caused broadening of the transition curve but did not significantly alter the phase transition temperature of the liposomes (*T*_*m*_ = 50.14°C) when compared to that of blank liposomes (*T*_*m*_ = 50.68°C). This suggests that resveratrol most likely integrated into the liposome membrane resulting in anisotropic melting of the membrane. Similar inferences on the localization of resveratrol have been reported by several groups [40–44] using different techniques. The presence of 5-fluorouracil, on the other hand, did not show any significant change in the peak morphology and was very similar to that of the blank liposomes and it also did not significantly change the *T*_*m*_ of liposomes (*T*_*m*_ = 50.47°C) when compared to that of the blank liposomes. Thus it can be inferred that 5-FU either does not localize into the lipid membrane and is encapsulated into the aqueous core or is very loosely bound with the membrane. The dual drug loaded liposomal nanoparticles showed a broader melting curve in comparison to the blank liposome sample. The *T*_*m*_ also was slightly reduced to 49.61°C when compared with the blank liposomes. It may be concluded that the localization of both drugs in the membrane induced anisotropic melting of the lipid bilayer.

### 3.4. Solid State NMR Studies

**Figure 4:**
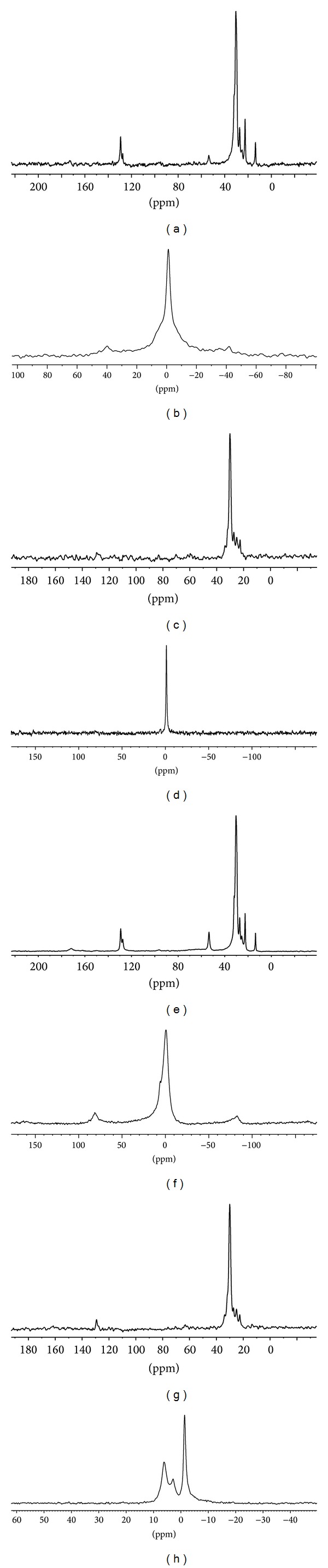
Solid state NMR spectra of (a) ^13^C spectra of blank liposomes; (b) ^31^P spectra of blank liposomes; (c) ^13^C spectra of resveratrol-loaded liposomes; (d) ^31^P spectra of resveratrol-loaded liposomes; (e) ^13^C spectra of 5-FU-loaded liposomes; (f) ^31^P spectra of 5-FU-loaded liposomes; (g) ^13^C spectra of dual drug loaded liposomes and (h) ^31^P spectra of dual drug loaded liposomes.

**Figure 5:**
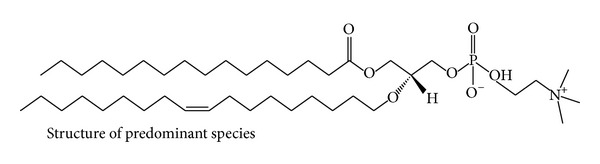
Structure of palmitoyl oleoyl phosphatidyl choline, the major constituent in the phospholipid used to form liposomes.

To further study the localization of the two molecules in the lipid membrane ^13^C and ^31^P solid state NMR studies were carried out. Solid state ^13^C and ^31^P NMR studies provide valuable information regarding the structure and the dynamics of the lipid membrane. The ^31^P NMR spectrum for liposome samples provides information on the size of the liposome, the phase in which the lipid molecules are present in, and any possible interaction between the drug molecule and the membrane [45]. These experiments were performed at room temperature (25°C).  Figure 4 shows the solid state ^31^P and ^13^C NMR spectra for the blank, resveratrol-loaded, 5-FU-loaded, and dual drug loaded liposomes. The structure of the major phospholipid constituent used for the formation of liposomes is shown in Figure 5. Apart from the phosphocholine groups, the palmitoyl oleoyl phosphatidyl choline also contains palmitic and oleic acid chains.

It was observed that the ^13^C spectra of blank liposomes exhibited a typical strong up-field shift of saturated palmitic acid chain at 13 to 30 ppm (Figure 4(a)). The C=C resonance was observed at 127 to 129 ppm and the choline head group was observed at 53 ppm [46]. The spectra for the 5-FU-loaded liposome sample showed the presence of all the peaks as observed in the blank liposome sample suggesting that 5-FU does not interact with the hydrophobic fatty acid region of the liposome bilayer (Figure 4(e)). These results are in agreement with those of El Maghraby et al., [47] who proved by calculation of polar surface areas that 5-FU localizes at the bilayer-water interface with some penetration into the lipid domain. Similarly, Okamura and Yoshii, [48] showed using ^1^H NMR that 5-FU binds loosely to the lipid membrane. In case of the resveratrol-loaded liposomes, the strong signal for aliphatic carbon chain is observed from 22 to 30 ppm but the other peaks for the choline head group at 53 ppm and the signal for the olefinic carbons are absent indicating that resveratrol localizes near the glycerol backbone of the lipid bilayer (Figure 4(c)). In the ^13^C spectra for the dual drug loaded liposome sample, the up-field methylenic shift is observed (22–29 ppm) (Figure 4(g)). However, the resonance peaks at 53 ppm, 127–129 ppm are absent indicating the localization of resveratrol in the lipid hydrophobic region. The absence of a clear peak at 127 to 129 ppm confirms that resveratrol localizes near the C=C region of the fatty acyl chain in the dual drug loaded liposomes.

**Figure 6:**
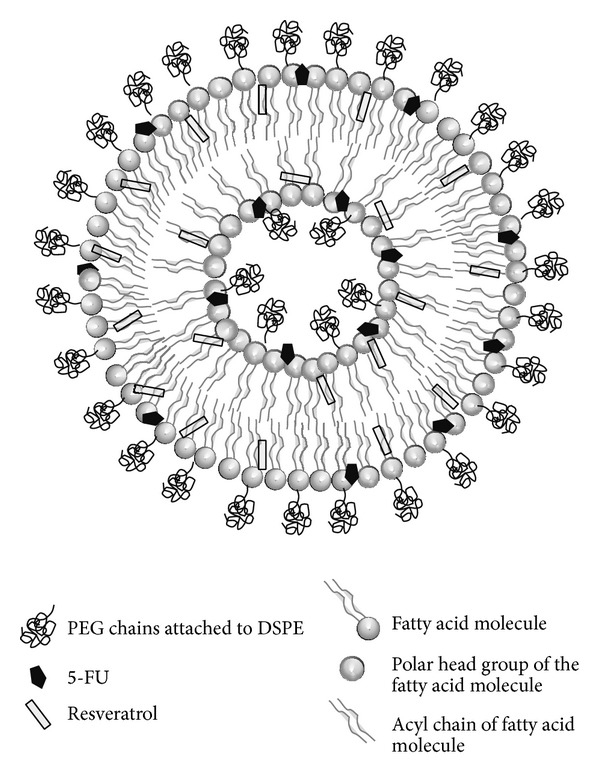
Diagrammatic representation of dual drug loaded PEGylated liposome.

The ^31^P NMR spectra for blank liposomes show a symmetric peak indicating the lamellar phase of the liposomes [44, 46] (Figure 4(b)). In the resveratrol-loaded liposomes this peak is considerably narrow signifying chemical isotropy in the bilayer brought about by incorporation of resveratrol (Figure 4(d)). In the 5-FU-loaded liposomes there was considerable broadening of the peak confirming that 5-FU localizes near the phosphate moiety, thus causing chemical shift anisotropy (Figure 4(f)). In the dual drug loaded liposomes there are two different peaks observed indicating the presence of two different interactions of phosphate moiety, one with the loosely bound 5-FU and the other with resveratrol, which induces isotropy in the membrane. Based on the DSC and NMR results, it may be inferred that the dual drug loaded PEGylated liposome has the following localization of the drugs as shown in Figure 6.

### 3.5. Release Kinetics

**Figure 7:**
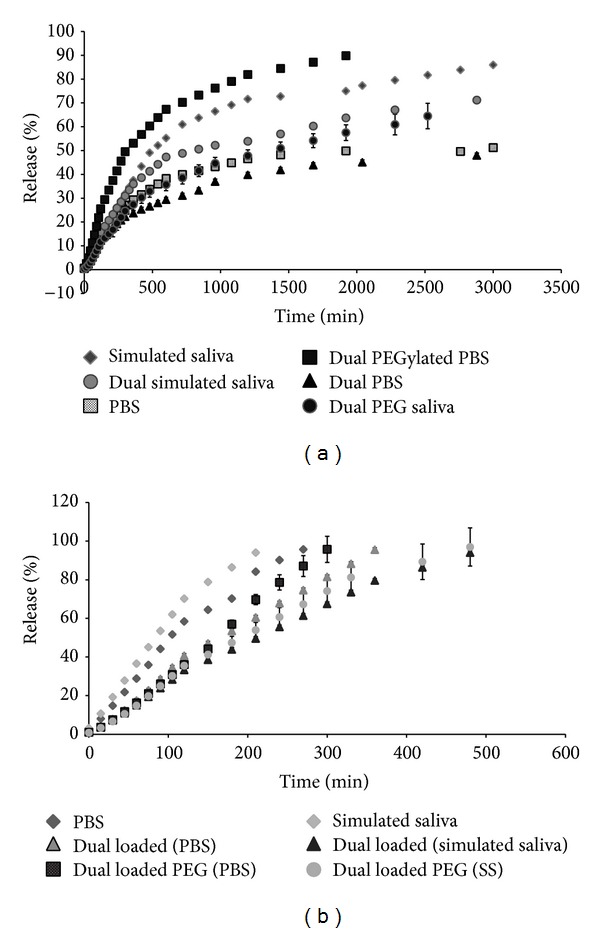
Release profiles of the (a) resveratrol and (b) 5-FU from the non-PEGylated and PEGylated dual drug loaded liposomes in different release medium at 37°C.

The localization of the two drugs in the membrane is one of the factors that are expected to influence their release from the liposome. This is demonstrated in our experiments wherein resveratrol located in the hydrophobic region of the membrane exhibits a slower and steady release while 5-fluorouracil located near the polar head group of the bilayer shows a faster release in both physiological pH 7.4 and the slightly acidic pH of 6.8 that is similar to that of saliva. Figures 7(a) and 7(b) show the release profiles of resveratrol and 5-FU from the single-loaded and dual drug loaded liposomes in PBS (phosphate buffered saline, pH 7.4) and simulated saliva medium (pH 6.8). The release from both PEGylated and non-PEGylated liposomes was recorded.

It was observed that the release profile of resveratrol from liposomes exhibited an initial burst release followed by a period of sustained release. The initial burst release may be attributed to the release of drug molecules that are present in the periphery of the nanocarrier. It was evident from the release pattern in simulated saliva medium that the release of resveratrol from the single drug loaded liposome was the quickest while the release rate is retarded in the presence of 5-fluorouracil in the dual drug loaded liposomes (Figure 7(a)). The release rates were further retarded in simulated saliva medium in the presence of PEG chains. In phosphate buffered saline, the release of resveratrol from the PEGylated dual drug loaded liposomes was found to be rapid when compared with its release from the single drug loaded and non-PEGylated counterparts in PBS. The difference in the release of resveratrol from the PEGylated liposomes in PBS and simulated saliva medium could arise due to differences in the conformation of the PEG chains in both systems. Though the mushroom-to-brush transitions of PEG chains have been generally attributed to the amount of PEG chains on the liposomal surface, other factors such as the surface charges and the packing parameter of the lipid bilayer have also been identified to influence the conformational transitions of the PEG chains [49]. The difference in the phosphate buffered saline and simulated saliva medium is in their pH and ion composition, which in turn can alter the hydration layer around the polar head groups and consequently affect the packing parameters of liposomal bilayer [50–52]. This may be the cause for the observed differences in the release rates of resveratrol from PEGylated liposomes in PBS and simulated saliva medium.

In the case of 5-FU, it was observed from the release patterns that a pronounced burst release is absent in the dual drug loaded liposomes. Also, the release of 5-FU is more than 10 times faster than the release of resveratrol under the same conditions. This suggests that the 5-FU is loosely bound and localized in the outer leaflet of the liposomal bilayer resulting in its rapid release. It was also found that the release of 5-FU in the presence of resveratrol is slowed by three times when compared to its release from single drug loaded liposomes. As observed with the release of resveratrol, the release of 5-FU from the PEGylated liposomes in PBS was faster than that observed in simulated saliva which may be attributed to the influence of the medium on the conformation of the PEG chains as well as packing parameter of the lipid bilayer. To understand the release profiles of the two drugs from the liposomal nanocarrier, model dependent kinetics was applied to the release data of each sample group. Four major mathematical models were applied, namely, zero order (% release versus time), first order (log_10%_ release versus time), Higuchi kinetics (% release versus square root time), and Korsmeyer Peppas (K-P) model (log % release versus log time). The release exponent (*n*) in the Peppas equation can be used to characterize the different release mechanisms. Briefly, *n* = 0.5 indicates Fickian diffusion pattern, 0.5 < *n* < 1.0 indicates non-Fickian or anomalous diffusion, that is, diffusion accompanied with slight erosion of the carrier, *n* = 1.0 indicates zero order or Case II transport mechanism, and *n* > 1.0 indicates super Case II transport [53]. The most suitable kinetic patterns were chosen from the highest *R* ^2^ value in each model. The *R* ^2^ values obtained for the release of resveratrol and 5-FU under different conditions are summarized in Table 4.

It was observed that the release of resveratrol from liposomes in PBS as well as simulated saliva medium followed both the Higuchi model of diffusion and the Korsmeyer-Peppas model. The curve fitting in the K-P model indicated Fickian diffusion for all sample groups irrespective of the release medium. This suggests that the release of resveratrol from the liposomes is governed by diffusion and erosion. It was also found that the release of 5-FU from the liposomes obeyed zero order kinetics in addition to the Higuchi and Korsmeyer-Peppas models. The curve fitting in the K-P model indicated a pattern slightly anomalous to Case II transport agreeing with the zero order steady kinetics. This can be explained by the localization of the drug molecule near the phosphate head group in the liposome membrane, which results in a fast release proportional to time. These results are in accordance with those of Glavas-Dodov et al. [54] who also proposed zero order kinetics for 5-FU loaded in liposomes and that liposomes act as a reservoir for steady release of 5-FU [54]. On applying curve fitting for the K-P model, it was found that the 5-FU release profile showed good correlation with the non-Fickian diffusion pattern.

### 3.6. GI_50_ (Growth Inhibition) Estimation and Drug Combination Analysis

**Figure 8:**
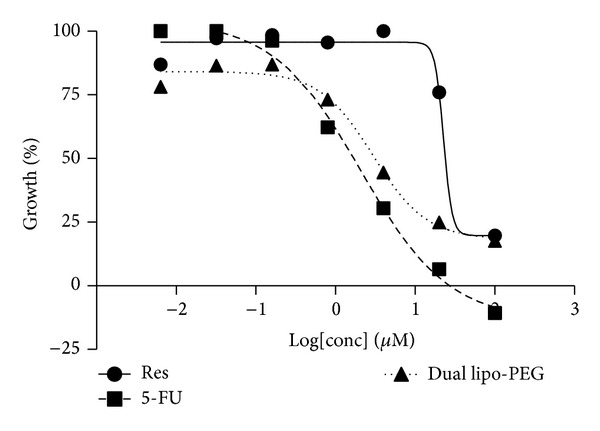
Dose response curves obtained for NT8e cells exposed to different concentrations of free resveratrol, 5-FU, and PEGylated dual drug loaded liposomes (Res—resveratrol, 5-FU—5-fluorouracil, and dual lipo-PEG—PEGylated dual drug loaded liposome).

Dose response curves for resveratrol and 5-fluorouracil in free and liposomal forms were estimated in NT8e oral cancer cell line.  Figure 8 shows the dose response curves for the NT8e cells exposed to different concentrations for free resveratrol, 5-FU, and the PEGylated dual drug loaded liposomes.

It was found that resveratrol has a higher GI_50_ (31 *μ*M) than 5-fluorouracil (2.2 *μ*M), which is an established chemotherapeutic agent. However, the dual drug liposomal formulation achieves 50% cell death at 2.6 *μ*M of 5-fluorouracil and 5.2 *μ*M of resveratrol. Blank liposome shows a GI_50_ of greater than 100 *μ*M and is comparable to the control, indicating no cytotoxicity. To determine the nature of combination of the two drugs, the median effect principle outlined by Chou [55] was utilized. The median effect principle is used to study drug interactions by plotting the dose-effect curves for each drug and either constant or nonconstant ratios of each molecule using the following median effect equation:

(2) $$\begin{array}{c} \frac{f_{a}}{f_{u}}={\left(\frac{D}{D_{m}}\right)}^{m}, \end{array}$$

where *f*_*a*_ is the fraction affected, *f*_*u*_ is the fraction unaffected, *D* is the dose of each drug, *D*_*m*_ is the dose for median effect (GI_50_ here), and *m* is the coefficient of sigmoidicity of the dose-effect curve [34, 56, 57]. The log version of the above equation gives the median effect plot where log⁡(*f*_*a*_/*f*_*u*_) is plotted on the *y*-axis and log⁡⁡(*D*) is plotted on the *x*-axis. “*m*” gives the slope of the curve and *D*_*m*_ denotes the antilog of *X* intercept. This is followed by the isobologram analysis devised by Chou and Talalay [34, 56, 57] giving the combination index (CI) for the two drugs using the equation:

(3) $$\begin{array}{c} \text{CI}=\left(\frac{D_{1}}{D_{X1}}\right)+\left(\frac{D_{2}}{D_{X2}}\right), \end{array}$$

where, *D*_*X*1_ is the dose of drug 1 required to produce the effect *X* and similarly *D*_*X*2_ is the dose of drug 2 required to produce the effect *X* individually. *D*_1_ is the dose of drug 1 required to produce the effect when used in combination with dose *D*_2_ of drug 2. Consider

(4) $$\begin{array}{c} D_{X}=D_{m}\left\lfloor \frac{f_{a}}{{\left(1-f_{a}\right)}^{1/m}}\right\rfloor . \end{array}$$

The combination index indicates whether the two drugs interact in an additive (CI = 1), synergistic (CI < 1), or antagonistic (CI > 1) manner [34, 56, 57].  Table 5 summarizes the CI grades assigned for different combinations of resveratrol and 5-FU. The grading for the CI values has been performed as suggested by Chou [34].

The median effect analysis and calculation of combination index of free resveratrol and 5-fluorouracil using the Compusyn software indicated that resveratrol and 5-fluorouracil produced different results at different concentrations (Table 5). Resveratrol at higher concentration (30 *μ*M) produces synergistic effect when used with 5-fluorouracil (0.5 *μ*M, 1.5 *μ*M, 2.5 *μ*M, and 5 *μ*M) while lower concentrations of resveratrol (3 *μ*M, 5 *μ*M, and 10 *μ*M) produce antagonistic effects. When the concentration of resveratrol is 15 *μ*M, a synergistic effect is observed at lower concentrations of 5-FU (≤1 *μ*M). This confirms that combination effects of two or more drugs are governed by their respective concentrations [58]. However, it is interesting to note that the ratio of resveratrol and 5-fluorouracil (5 *μ*M for resveratrol and 2.5 *μ*M of 5-fluorouracil) produced strong antagonistic effect. The same ratio of both drugs is present in the dual drug loaded nanoformulation. In an attempt to understand the influence of the nanocarrier on the combination effects of resveratrol and 5-FU, an* in vitro* study was performed to determine the cytotoxic potential of the combination of individually loaded resveratrol liposomes and 5-fluorouracil liposomes (Table 6).

It was observed that at low concentration of both formulations, the drugs acted in an antagonistic manner.

However, on increasing the concentration of resveratrol to 20 or 30 *μ*M the interaction was synergistic to most concentrations of 5-fluorouracil (0.25 *μ*M, 0.5 *μ*M, 1 *μ*M, and 2 *μ*M). Synergistic effect was also observed even at resveratrol concentrations of 10 *μ*M in combination with 0.5 *μ*M or 1 *μ*M concentration of 5-FU. This suggests that encapsulation of the drugs in liposomes may have an influence in enhancing their synergistic interactions. This aspect opens new avenues for designing nanocarrier-based multidrug chemotherapy regimens in the future. It was observed that concentrations of liposomal resveratrol (5 *μ*M) and liposomal 5-FU (2.5 *μ*M) exhibit moderate antagonism, a slight improvement over the free drugs which at the same concentrations exhibited strong antagonism (Table 6).

### 3.7. Gene Expression Studies

**Figure 9:**
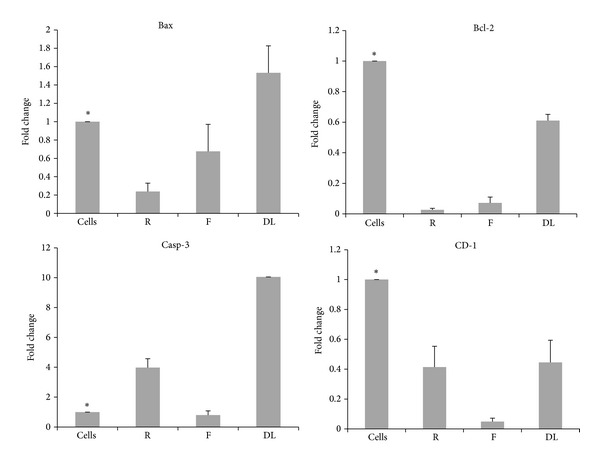
Expression levels of bax, bacl-2, caspase-3, and cyclin D1 in cells treated with resveratrol (31 *μ*M), 5-FU (2.6 *μ*M), and PEGylated dual drug loaded liposomes (containing 2.5 *μ*M of 5-FU and 5 *μ*M of resveratrol); R—resveratrol, F—5-FU and DL—dual drug loaded PEGylated liposomes, and cells—untreated cells. ^∗^signifies that the groups are significantly different from the DL group (*P* < 0.05).

Analysis of gene expression offers an indication as to the signaling pathway, which might be followed by this formulation in inducing apoptosis. The levels of antiapoptotic gene (*bcl-2*), proapoptotic gene* bax* and levels of* caspase-3*, an executer of the caspase cascade, and* cyclin D1,* an important regulator of the transition from G1 to S phase of the cell cycle, have been evaluated (Figure 9). The cells were treated with free resveratrol (31 *μ*M) or free 5-FU (2.6 *μ*M) or dual drug loaded PEGylated liposomes (containing 2.5 *μ*M of 5-fluorouracil and 5 *μ*M of resveratrol). It was found that the levels of* bax* in the treated cells were significantly higher than the untreated control cells and levels of* bcl-2* were lower than those of the untreated cells. From the expression levels, it can be inferred that the dual drug loaded PEGylated liposomes exhibit differential effects on different genes. The expression level of the proapoptotic gene* bax* was upregulated in the case of the PEGylated dual drug loaded liposomes while it remained downregulated when treated with free resveratrol and free 5-FU. This indicates that the drugs might exert a synergistic effect when present together in the liposomal carrier. The levels of antiapoptotic gene* bcl-2* were downregulated in all the treated groups. However, the magnitude of downregulation was maximum in the case of free resveratrol and free 5-FU. This suggests that the drugs when coencapsulated in the liposome exhibit an antagonistic effect on the expression levels of* bcl-2*. The expression levels of* caspase-3* were upregulated in the treated groups with the maximum upregulation observed with the dual drug loaded liposomes suggesting a synergistic action of the drugs when coencapsulated in the liposome on* caspase* expression. The levels of* cyclin D1* in the dual drug loaded liposomes though downregulated were higher than those of the 5-fluorouracil treated cells, hence indicating some antagonism at these concentrations of the two drugs. The results of the gene expression levels indicate that the coencapsulation of resveratrol and 5-FU in liposomes exerts different combination effects on different genes. It can be inferred that though the important markers of apoptosis like* bcl-2*,* bax* levels, and* caspase-3* activation and lowering of* akt* levels seem in agreement with cytotoxicity, there might be other mechanisms that may contribute to the antagonistic effect of the combination of the two drugs.

## 4. Conclusion

This work depicts the successful development and characterization of a dual drug loaded liposomal nanoformulation. The interaction of resveratrol and 5-fluorouracil in a liposomal carrier administered to head and neck cancer cell line was studied for the first time. The combined action of the two drugs at the GI_50_ was found to be slightly antagonistic and not synergistic when analysed using the median effect principle. Coencapsulation of the drugs in the liposomal carrier seems to reduce the threshold concentrations required for synergistic effects when compared with the free drug combinations. The expression levels of cells* bcl-2*,* cyclin D1*, and* akt* showed desired downregulation whereas* bax*,* caspase-3* showed upregulation of genes indicating apoptosis when treated with the liposomal formulation. The liposomal carrier induces differential combination effects on the expression of genes and the net effect of these effects may influence their cytotoxicity.

## Figures and Tables

**Table 1:** Primers list.

Gene name	Forward sequence	Reverse sequence
*GAPDH *	CGGATTTGGTCGTATTGG	AGTAGAGGCAGGGATGATG
*bcl 2 *	TGCGGCCTCTGTTTGATTTC	GGCATGTTGACTTCACTTGTGG
*bax *	CTTACGTGTCTGATCAATCCCC	ACTTGAGCAATTCCAGAGGC
*Caspase-3 *	TCATACCTGTGGCTGTGTATCC	TGAGTTTTCAGTGTTCTCCATGG
*Cyclin D1 *	GTGAACAAGCTCAAGTGGAACC	TGATCTGTTTGTTCTCCTCCGC

**Table 2:** Optimization of encapsulation efficiency for liposomal 5-FU.

Drug : lipid ratio (w/w)	Encapsulation efficiency (%)
1 : 1	3.56 ± 1.20^a^
2 : 5	3.20 ± 0.66^a^
4 : 5	3.73 ± 0.51^a^
1 : 10	4.00 ± 0.58^a^
1 : 20	14.76 ± 3.33
1 : 30	9.68 ± 2.32
1 : 40 : 8∗	5.29 ± 0.06^b^
1 : 20 : 1∗	15.52 ± 0.92
1 : 40 : 1∗	9.31 ± 0.63^b^
1 : 40 : 2∗	15.25 ± 0.15
1 : (18 : 2)∗∗	5.81 ± 0.95^c^
1 : 1 : (18 : 2)∗∗∗	24.63 ± 1.46

^a^denotes that the groups are significantly different from the 1 : 20 ratio (*P* < 0.05).

^b^denotes that the groups are significantly different from the 1 : 40 : 2 ratio (*P* < 0.05).

^c^denotes that the groups are significantly different from the 1 : 1 : (18 : 2) ratio (*P* < 0.05).

∗denotes 5-FU : EPC : Res (dual encapsulated formulation without DSPE-PEG).

∗∗denotes 5-FU : EPC : DSPE-PEG (PEGylated 5-FU formulation).

∗∗∗denotes 5-FU : Res : EPC : DSPE-PEG (PEGylated dual liposomal formulation).

**Table 3:** Optimization of encapsulation of liposomal Res.

Drug : lipid ratio (w/w)	Encapsulation efficiency (%)
1 : 10	20.93 ± 1.83^a^
1 : 20	18.93 ± 0.6244^a^
1 : 40	52.36 ± 2.71
1 : 60	53.74 ± 10.17
1 : 40 : 8∗	35.98 ± 5.52
1 : 20 : 1∗	20.21 ± 2.7^b^
1 : 40 : 1∗	27.14 ± 2.97
1 : 40 : 2∗	33.46 ± 1.91
1 : (38 : 2)∗∗	26.55 ± 4.85
1 : (18 : 2)∗∗	38.91 ± 2.58^c^
1 : 1 : (18 : 2)∗∗∗	41.56 ± 0.695^c^

^a^denotes that these groups are significantly different from 1 : 40 ratio (*P* < 0.05).

^b^denotes that the group is significantly different from the 1 : 40 : 2 ratio (*P* < 0.05).

^c^denotes that the group is significantly different from the 1 : 38 : 2 ratio (*P* < 0.05).

∗denotes Res : EPC : 5-FU (dual encapsulated formulation without DSPE-PEG).

∗∗denotes Res : EPC : DSPE-PEG (PEGylated Res formulation).

∗∗∗denotes 5-FU :  Res : EPC : DSPE-PEG (PEGylated dual liposomal formulation).

**Table 4:** Regression coefficients obtained using different kinetic models for the release of resveratrol and 5-FU from different liposomes.

Drug	Release condition	Zero order (*R* ^2^)	First order (*R* ^2^)	Higuchi model (*R* ^2^)	Peppas model	
					(*R* ^2^)	*n*
Res	PBS, pH 7.4	0.654	0.358	0.880	**0.937 **	0.462
	Simulated saliva, pH 6.8	0.778	0.447	0.932	**0.950 **	0.412
	Dual loaded liposomes in PBS (pH 7.4)	0.782	0.430	**0.957 **	**0.962 **	0.436
	Dual loaded liposome in simulated saliva (pH 6.8)	0.768	0.413	0.939	**0.952 **	0.443
	Dual loaded PEGylated liposomes in PBS (pH 7.4)	0.790	0.427	**0.946 **	**0.955 **	0.458
	Dual loaded PEGylated liposomes in simulated saliva (pH 6.8)	0.870	0.466	**0.979 **	**0.957 **	0.529

5-FU	PBS (pH 7.4)	**0.976**	0.711	0.956	**0.974**	0.772
	Simulated saliva (pH 6.8)	**0.971 **	0.735	0.95	**0.972 **	0.758
	Dual loaded liposomes in PBS (pH 7.4)	**0.987 **	0.708	0.958	**0.967 **	0.846
	Dual loaded liposomes in simulated saliva (pH 6.8)	**0.985 **	0.692	0.965	**0.971 **	0.811
	Dual loaded PEGylated liposomes in PBS (pH 7.4)	**0.992 **	0.751	0.900	**0.958 **	1.014
	Dual loaded PEGylated liposomes in simulated saliva (pH 6.8)	0.954	0.614	0.968	**0.974**	0.753

**Table 5:** Combination index (CI) of different resveratrol and 5-FU combinations *in  vitro* and grading as per the literature [Chou, 2006 [34]].

Resveratrol (*μ*m)	5-FU (*μ*m)	CI grade
3	0.5	± (nearly additive)
	1.5	− − − (antagonism)
	2.5	− − − − (strong antagonism)
	5	− (slight antagonism)

5	0.5	− − − (antagonism)
	1.5	− − − − (strong antagonism)
	2.5	− − − − (strong antagonism)
	5	− − (moderate antagonism)

10	0.5	− − − (antagonism)
	1.5	− − − − (strong antagonism)
	2.5	− − − − − (very strong antagonism)
	5	− − − (antagonism)

15	0.25	+ + + (synergism)
	0.5	+ + (moderate synergism)
	1	+ (slight synergism)
	2	− − (moderate antagonism)
	4	− − − (antagonism)

30	0.25	+ + (moderate synergism)
	0.5	+ + (moderate synergism)
	1	− (slight antagonism)
	1.5	+ + + + (strong synergism)
	2	− − − (antagonism)
	2.5	+ + + (synergism)
	5	+ + + (synergism)

**Table 6:** Combination index (CI) of individually loaded resveratrol liposomes and 5-FU liposomes.

Res (*μ*m)	5-FU (*μ*m)	CI grade
5	0.25	− − − − − (very strong antagonism)
	0.5	− − − − − (very strong antagonism)
	1	− − − − − (very strong antagonism)
	2	− − (moderate antagonism)
	5	− − (moderate antagonism)

10	0.25	− − − − − (very strong antagonism)
	0.5	+ + (moderate synergism)
	1	+ + + (synergism)
	2	− (slight antagonism)
	5	− (slight antagonism)

20	0.25	− − − − − (very strong antagonism)
	0.5	+ + + + (strong synergism)
	1	+ + + (synergism)
	2	+ + (moderate synergism)
	5	± (nearly additive)

30	0.25	+ + + + (strong synergism)
	0.5	+ + + + (strong synergism)
	1	+ + + (synergism)
	2	+ + + (synergism)
	5	+ + (moderate synergism)
